# Testing of Automated Photochemical Reflectance Index Sensors as Proxy Measurements of Light Use Efficiency in an Aspen Forest

**DOI:** 10.3390/s18103302

**Published:** 2018-10-01

**Authors:** Saulo Castro, Arturo Sanchez-Azofeifa

**Affiliations:** Earth and Atmospheric Sciences, University of Alberta, Edmonton, AB T6G-2E3, Canada; scastro@ualberta.ca

**Keywords:** remote sensing, PRI, LUE model, eddy covariance

## Abstract

Commercially available autonomous photochemical reflectance index (PRI) sensors are a new development in the remote sensing field that offer novel opportunities for a deeper exploration of vegetation physiology dynamics. In this study, we evaluated the reliability of autonomous PRI sensors (SRS-PRI) developed by METER Group Inc. as proxies of light use efficiency (LUE) in an aspen (*Populus tremuloides*) forest stand. Before comparisons between PRI and LUE measurements were made, the optical SRS-PRI sensor pairs required calibrations to resolve diurnal and seasonal patterns properly. An offline diurnal calibration procedure was shown to account for variable sky conditions and diurnal illumination changes affecting sensor response. Eddy covariance measurements provided seasonal gross primary productivity (GPP) measures as well as apparent canopy quantum yield dynamics (α). LUE was derived from the ratio of GPP to absorbed photosynthetically active radiation (APAR). Corrected PRI values were derived after diurnal and midday cross-calibration of the sensor’s 532 nm and 570 nm fore-optics, and closely related to both LUE (*R*^2^ = 0.62, *p* < 0.05) and α (*R*^2^ = 0.72, *p* < 0.05). A LUE model derived from corrected PRI values showed good correlation to measured GPP (*R*^2^ = 0.77, *p* < 0.05), with an accuracy comparable to results obtained from an α driven LUE model (*R*^2^ = 0.79, *p* < 0.05). The automated PRI sensors proved to be suitable proxies of light use efficiency. The onset of continuous PRI sensors signifies new opportunities for explicitly examining the cause of changing PRI, LUE, and productivity over time and space. As such, this technology represents great value for the flux, remote sensing and modeling community.

## 1. Introduction

The light use efficiency (LUE) model [1,2] has provided an avenue for quantifying the terrestrial carbon cycle using remote sensing data [3,4]. The LUE model can be conceptualized as being driven by structural and pigment pool changes in vegetation and, secondly, by the physiological status described by the vegetation’s photosynthetic process [5]. The structural component is quantified by the amount of photosynthetically active radiation absorbed by the canopy (APAR); while the physiological component is described as the efficiency by which the absorbed light, or light use efficiency (LUE), is used to fix carbon during a specific period. Much work has been done in displaying a relationship between optical remote sensing and the APAR term, although it is not without error and complexity [6,7,8,9,10,11]. However, as LUE variability can be driven by multiple individual and combined factors, the connection between efficiency and remote sensing signals have been harder to characterize, leading to considerable uncertainty in many ecosystems [12,13,14]. Some of the physiological effects on light use efficiency include changing climatic conditions, temperature, water availability, phenology, vegetation functional type, and vegetation species [15,16,17,18,19,20,21,22]. These complex interactions confound the parametrization of in LUE in global productivity models, and, as such, efforts continue towards the better accounting of efficiency changes that would, in turn, lead to more accurate CO_2_ flux estimations.

Developments in proximal and spaceborne remote sensing have led to advances in estimating vegetation LUE at the leaf level, stand level, and whole ecosystem resolutions [14,23,24,25]. One of the main proposed tools to do so is through the use of the photochemical reflectance index (PRI). First developed in leaf level studies, this vegetation index has been shown to be a proxy of xanthophyll pigment activity and closely linked to photosystem II (PSII) photochemical efficiency [23,26,27]. During periods of light saturation and excess energy PSII centers remain in a reduced state, leaving them susceptible to photoinhibitory damage [28]. Non-photochemical quenching (NPQ) performed through the chemical transformation of carotenoid pigments that compose the xanthophyll cycle prevent damage to photosynthetic centers. More specifically, NPQ of excess light is done through the de-epoxidation of violaxanthin to the photoprotective pigment zeaxanthin, with antheraxanthin acting as a transitional pigment [28,29,30]. De-epoxidation provides a sink for excess energy and a method of light regulation that can be measured optically via the PRI [27,31,32]. PRI tracks short-term decreases in reflectance at 531nm that occur in response to increased zeaxanthin concentrations and shrinking chloroplast (associated with increased thylakoid pH) resulting from xanthophyll de-epoxidation [23,27]. The index uses reflectance at 570 nm (insensitive to NPQ) as a reference band. 

Testing of PRI over seasonal timescales for foliar, canopy and ecosystem level scales has shown PRI to be a good indicator of physiological efficiency across different vegetation types and scales, however, with numerous temporal and spatial challenges [24]. Radiative transfer modeling studies have identified a number of confounding factors including leaf area distribution, soil type, and view and illumination angles affect the interpretation of PRI [33,34]. Additionally, in situ studies have shown a strong effect of chlorophyll/carotenoid ratios on seasonal PRI [24]. These changes in pigment pool sizes can account for a significant proportion of seasonal PRI (constitutive) changes, compared to those driven by xanthophyll cycle activity (facultative changes), and lead to misrepresentations of the PRI-photosynthetic relationship [19,35,36,37,38]. As such, seasonal studies are needed to characterize the effects of vegetation structure changes and identify the facultative and constitutive components in PRI [39]. However, few long-term studies with the necessary dense time-series measurements over varying ecosystems exist, and some of these rely on satellite-based data with low temporal resolutions that do not allow the separation of facultative and constitutive effects. Perhaps for this reason, some comparisons of satellite-based PRI and LUE across ecosystems have resulted in contrasting PRI-LUE relationships for different ecosystems [40,41]. 

The lack of accessible and cost-effective data, available at meaningful temporal or spatial scales, has limited the testing of PRI functionality at ecosystem levels. For example, a 16-day MODIS composite lacks the temporal resolution to resolve short-term xanthophyll cycle changes. This has contributed to slow integration and comparison to micrometeorological data and ecosystem monitoring networks [42]. However, the recent availability of inexpensive PRI specific narrowband radiometers [43,44,45] has opened the possibility of attaining continuous PRI measurements at low cost. The high temporal resolution of automated optical sensor systems would allow improved accounting of physical and physiological variables affecting diurnal and seasonal PRI measurements and help interpret LUE changes over a phenological cycle. Additionally, continuous PRI measurements allow for their integration with eddy covariance flux measurements, which in turn provides additional ecophysiological validation for PRI as an LUE proxy at ecosystem scales.

For this study, we utilized newly commercially developed automated PRI spectral reflectance (SRS-PRI) radiometers and eddy covariance data to explore the LUE dynamics in a deciduous boreal forest. As such, the objectives of this paper are to (1) determine if automated SRS-PRI values can be used as a proxy of canopy light use efficiency and apparent canopy quantum yield over an aspen forest and (2) to explore how continuous PRI measurements can be leveraged to better inform ecosystem scale LUE models.

## 2. Materials and Methods

### 2.1. Study Area

**Figure 1:**
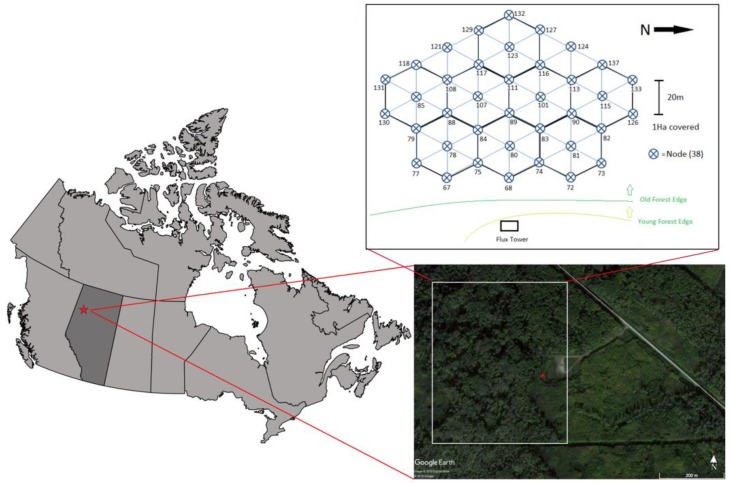
Location of the Peace River Environmental Monitoring Super Site (red star). Red triangle shows the location of the flux tower at the edge of an old growth deciduous boreal forest. The tower contains eddy covariance system, meteorological sensors, and proximal remote sensing sensors. A Wireless Sensor Network (WSN) composing of 36 nodes is located east of the eddy covariance tower, within the flux footprint.

The study was conducted at the Peace River Environmental Monitoring Super Site (PR-EMSS) within the Ecosystem Management Emulating Natural Disturbance (EMEND) site, located approximately 90 km northwest of Peace River, AB, Canada (Figure 1). This region is part of the Lower Foothills Natural Subregion [46]. The study plot, located at 56°44′38.10″ N, 118°20′38.08″ W, with an altitude of 867 m ASL, is characterized as an old growth stand of trembling aspen (*Populus tremuloides*). There are two vertical vegetation layers; the understory reaching a height of 3 m, while the overstory canopy growing to 12–15 m. Its topography is slightly sloping (<2%) in the west direction. The predominant wind direction is from the west. Mean annual air temperature is 0.6 °C and mean annual precipitation is 436.2 mm, with approximately 29% falling as snow [47]. Soils are primarily Orthic and Dark Gray Luvisols and have a depth of 1 m on average [48]. The study site has been monitored since 2013 with an eddy covariance system and meteorological station installed on a 30 m tower located at the eastern edge of the forest stand. Proximal remote sensing sensors, comprising of PRI and broadband spot radiometers mounted on the tower allowed the tracking of forest stand phenology, as well as monitoring the incoming and canopy reflected light. A wireless sensor network (WSN) continually measured the transmitted photosynthetic photon flux density (PPFD) within the canopy, providing continuous measurements of fraction of absorbed photosynthetically active radiation (fAPAR).

### 2.2. Reflectance Measurements and Vegetation Indices

#### 2.2.1. Spectral Reflectance Sensors (SRS)

Spectral data was collected through the use of commercial automated SRS-PRI sensors with an Em50 datalogger (METER Group. Inc., Pullman, WA, USA). PRI sensors contain photodiodes with interference filters at selected peak wavelengths 532 ± 2 and 570 ± 2 nm with 10 nm full width half maximum (FWHM) bandwidths. Photodiode and filter construction was based on prototypes reviewed by Garity et al. [43]. Downward looking PRI pairs contain field stops, restricting the field of view (FOV) at 36°. Upward looking pairs contain Teflon cosine diffusers allowing near 180° FOV. They provide continuous irradiance measurements used to normalize radiance spectral responses measured through the downward looking sensor pair. Upwelling and downwelling PRI sensors were placed on a 25 m tower and positioned 10° off-nadir at an approximate distance of 10–12 m from the top of the canopy, representing a footprint of approximately 50 m^2^ towards the eddy covariance area of influence. 

Each sensor produced a radiance and irradiance output, for each waveband (532 nm and 570 nm) at 1-minute intervals throughout the 2015 season. Data points were filtered to remove precipitation events which caused significant signal noise attributed to water droplets accumulating on the flat Teflon cosine diffusers of the hemispherical sensor pair. Uncorrected reflectance at each waveband was expressed as the ratio of measured radiance (*r*) to irradiance (*i*) (Equations (1) and (2)) and used to calculate uncorrected PRI (Equation (3)):

(1) $$\;\rho_{532}=\;\frac{r_{532}}{i_{532}}\;$$

(2) $$\;\rho_{570}=\frac{r_{570}}{i_{570}}\;$$

(3) $$\;\mathrm{PRI}=\frac{\rho_{532}-\rho_{570}}{\rho_{532}+\rho_{570}}\;$$

where ρ represents reflectance at a specific wavelength. Calculated PRI values were averaged over 30 min to match eddy covariance binned measurements. PRI values were expressed as scaled PRI (sPRI) using the formula [49,50]:

(4) $$\;\mathrm{sPRI}=\frac{\left(1+\mathrm{PRI}\right)}{2}\;$$

which transformed PRI values into a 0–1 range commonly used in remote sensing vegetation indices. Before and after field deployment, the sensors underwent a series of cross-calibration and sensor response validation experiments. Details of these experiments are outlined in Section 2.2.2 and Section 2.2.3 below.

#### 2.2.2. SRS-PRI Sensor Cross-Calibration

A cross-calibration procedure is required to account for differences in sensor response and fore-optics [51]. The cross-calibration process allows for the normalization of irradiance and radiance outputs under varying light conditions. Gamon et al. [44] outlined a procedure for both midday and diurnal cross-calibration, noting that diurnal calibrations best represent xanthophyll cycle epoxidation states. Due to the remoteness of our study site and the manual nature of cross-calibration, both the midday and diurnal cross-calibration procedures were done prior and subsequently to field deployment, following the general principles outlined by Gamon et al. [44].

SRS-PRI sensors were placed at the height of 30 cm over a 99% reflective white standard panel (Spectralon, Labsphere Inc., North Sutton, NH, USA), ensuring that the downward-looking sensor’s FOV covered the standard. The upward-looking sensor simultaneously monitored the sky’s irradiance conditions. The sensor pair measured continuously over a 23-day period. A pyranometer (SP-110, Apogee Instruments Inc., Logan, UT, USA) logged on a wireless sensor node (ENV-LINK-MINI, LORD Microstrain® Sensing Systems, Williston, VT, USA) was used to monitor incident radiation, sampled at 1-minute intervals. This radiation data was used to characterize the various illumination conditions over the experimental period, which varied from overcast to clear and sunny conditions. A solar radiation calculator (SolRad), developed by the Washington State Department of Ecology, provided modeled global radiation on a horizontal surface using the Ryan-Stolzenbach model [52]. Percent illumination was derived by comparing modeled to measured radiation data throughout the experiment. The 23-day experimental period allowed us to capture a nearly complete spectrum of illumination conditions (15–100%) over a full set of diurnal solar elevations (0°–56°). Solar elevations, defined as the angle between the horizon and the sun, were determined using NOAA’s solar position calculator (https://www.esrl.noaa.gov/gmd/grad/solcalc/). 

Cross-calibration responses as a function of illumination were calculated for every 3° solar elevations bin throughout the diurnal range. Three-degree bins were chosen as they represented approximately 30 min time intervals during the morning and evening periods where solar elevation changed most rapidly. A set of cross-calibration functions were derived for each sensor waveband. Percent illumination and solar elevations were calculated for onsite data collected during the 2015 season. Cross-calibration ratios derived from the set of empirical calibration functions were used to calculate corrected reflectance ($\rho_{corrected}$) as follows:

(5) $$\begin{array}{ll} \;\rho_{corrected} & =\frac{\rho_{uncorrected}}{{}^{\prime }Cross-calibration\;ratio^{\prime }}\\ & =\frac{r_{\mathrm{target}}/i_{\mathrm{sky}}}{r_{\mathrm{standard}}/i_{\mathrm{sky}}}\; \end{array}$$

where r_target_ represents radiance from the canopy, r_standard_ represents radiance from the white standard, and i_sky_ represents irradiance from sky conditions. Midday cross-correlation functions were also derived for each sensor waveband and used to calculate midday corrected reflectance following Equation (5). Diurnal and midday corrected reflectance were used to calculated (diurnal and midday) corrected PRI following Equation (3). Corrected PRI values were averaged over 30 min to match eddy covariance data.

#### 2.2.3. Sensor Response Validation

A dual-channel field spectrometer (Unispec-DC, PP-Systems, Amesbury, MA, USA) was used to validate the spectral response of the SRS-PRI sensors independently. Additionally, diurnal spectrometer measurements were collected in concert with cross-calibration data to confirm the effect and accuracy of mid-day vs. diurnal cross-calibration efforts. The Unispec-DC is a high precision spectrometer able to provide simultaneous measurements of radiance and irradiance, has a spectral range of 310–1100 nm, and a 3.3 nm FWHM. The upwelling/radiance was collected using a 2 m fiber optic cable (600 µm HCS LOH, SMA-Custom Ferrule-100 mm, Uni-684, PP Systems) with a 9 mm FOV restrictor providing a modified FOV of 18°. For the downwelling/irradiance, a similar 2 m fiber optic cable was used (600 µm HCS LOH, SMA-Custom Ferrule-25 mm, Uni-686, PP Systems) with the addition of a cosine head (UNI435, PP Systems) allowing near 180° FOV. 

Validation experiments consisted of the sampling the diurnal response of a ~1 m tall *Populus tremuloides* seedling stand, simultaneously, with the field spectrometer and SRS sensors, throughout a 5-day period. The spectrometer’s radiance fore-optic and SRS-PRI field-of-view pair were placed at matching height (0.37 m) above the canopy, producing a target footprint of about 1.0 m^2^. The SRS-PRI sensors log every minute continuously throughout the diurnal cycle; while the spectrometer measurements were collected every minute for 10 min on the hour throughout the diurnal cycle. 

Spectrometer reflectance data was corrected using a 99% reflective white standard panel (Spectralon, Labsphere Inc.). Corrected reflectance was expressed as:

(6) $$\;\rho_{corrected}=\frac{r_{\mathrm{target}}}{i_{\mathrm{sky}}}\;\times \frac{i_{\mathrm{sky}}}{r_{\mathrm{standard}}}\;\;$$

where *ρ*_corrected_ represents corrected reflectance. The first term (r_target_/i_sky_) represents the raw reflectance, expressed as a ratio of the upwelling radiance to the downwelling irradiance over the target. The second term (i_sky_/r_standard_) represents the cross-calibration value, calculated as a ratio of the downwelling irradiance to the radiance of the standard panel [44]. Corrected reflectance at 531 nm and 570 nm was used to derive a scaled photochemical reflectance index (sPRI). Corrected sPRI measurements from SRS-PRI sensors were compared to the spectrometer derived sPRI to validate the accuracy of these automated sensors.

### 2.3. Broad Band Optical Sensors and Wireless Sensor Network (WSN)

A wireless sensor network (WSN), composing of 36 nodes, was used to measure the fraction of absorbed photosynthetically active radiation (*f*APAR). The WSN sensor nodes were arranged in a hexagonal pattern, spaced every 20 m over a one-hectare plot located within the flux footprint. The spatial extent of the WSN footprint accounted for approximately 55% of measured flux measurements. Additional details on the WSN deployment and technical capabilities can be found in Rankine et al. [53]. Individual nodes were outfitted with an upward and downward facing quantum sensors (SQ-110, Apogee Instruments Inc.), providing transmitted PPFD and soil reflected PPFD measurements, respectively. Additional nodes were placed on the flux tower, positioned above the canopy. Upward and downward looking quantum sensor pairs provided measurements of incident and reflected PPFD, respectively. The sampling interval of WSN nodes was 15 min. *f*APAR was calculated using the equation:

(7) $$f\mathrm{APAR}=1-t-r+(t\times r_{s})$$

where *t* is the fraction of transmitted radiation, *r* is the reflected radiation from the canopy, and *r_s_* is the soil reflectance component.

A broadband normalized difference vegetation index (NDVI) was derived from upward and downward looking quantum and pyranometer pairs installed on the flux tower, positioned above the canopy. Spot radiometers had a sampling interval of 10 min. Broadband NDVI was calculated using the equation:

NDVI = (*ρ*PYR − *ρ*PPFD)/(*ρ*PYR + *ρ*PPFD) (8)

where *ρ*PYR is the solar radiation reflectance calculated from the upwelling:downwelling radiation from pyranometer pairs; and *ρ*PPFD is the total reflectance of PPFD derived from upwelling: downwelling PPFD sensor pairs. Broadband NDVI values have been shown as adequate proxies of narrowband NDVI measurements [54]. Seasonal NDVI values were used to identify phenologic stages. A second derivative function was used to identify the transitions from maturity to leaf senescence (21 August 2015).

### 2.4. Light Use Efficiency Calculations

LUE was derived following traditional remote sensing methodology through the rearrangement of the LUE model as follows:

(9) $$\mathrm{LUE}=\frac{\mathrm{GPP}}{\left(f\mathrm{APAR}\times \mathrm{PPFD}\right)}=\frac{\mathrm{GPP}}{\mathrm{APAR}}\;$$

where GPP is the gross primary productivity, derived from eddy covariance measurements, and the absorbed photosynthetically active radiation (APAR) is the product of *f*APAR and PPFD. LUE values were derived at both diurnal and seasonal time scales. LUE values were expressed as 30 min. binned averages. 

### 2.5. Micrometeorology Measurements

#### 2.5.1. Micrometeorology Instrumentation and Processing

The eddy covariance (EC) method [55,56,57] was used to measure net ecosystem CO_2_ exchange (NEE) (µmol m^−2^ s^−1^), (latent heat (LE) (W m^−2^), and sensible heat (H) (W m^−2^) fluxes during the 2016 growth cycle. This consisted of a fast response (20 Hz) closed-path infrared gas analyzer (IRGA) (model EC155, Campbell Scientific Inc., Logan, UT, USA) and a three-dimensional sonic anemometer (model CSAT-3A, Campbell Scientific Inc.). The system was installed on a scaffold tower at 25 m above the forest floor. The sonic anemometer and IRGA share integrated electronics and communications to reduce logging lag, as part of the CPEC200 EC system (Campbell Scientific). Both sensors have a sampling frequency of 20 Hz, logged with a CR3000 datalogger (Campbell Scientific, Logan). A complete weather station (model HOBO U-30-NRC Weather Station, Onset Computer Corp., Bourne, MA, USA) provide ancillary meteorological variables including temperature (T_air_), relative humidity (RH), vapor pressure deficit (VPD), precipitation, soil temperature, soil volumetric water content (VWC), soil heat fluxes, and net radiation. Remote communication to all micrometeorological instrumentation was provided through an Iridium satellite connection (Upward Innovations Inc., East Falmouth, MA, USA).

High-frequency eddy covariance data was processed using EddyPro^®^ software (LI-COR Inc., Lincoln, NE, USA) and IBM streams^®^ (IBM, Armonk, NY, USA). Vertical CO_2_ flux (NEE) (µmol m^−2^ s^−1^) was expressed as the product of mean air density and the covariance between instantaneous vertical wind velocity and concentration fluctuations.

(10) $$\mathrm{NEE}=-\rho_{a}\bar{w^{\prime }s^{\prime }}$$

where *ρ*_a_ is the dry air density (mol m^−3^), *w* is the instantaneous vertical wind speed (m s^−1^), and *s* is the molar mixing ratio (mol mol^−1^ dry air). The negative sign follows meteorological notation, where negative NEE values represented net CO_2_ uptake into an ecosystem, and positive values represent net CO_2_ release into the atmosphere.

Eddy covariance data corrections included time lag correction [58], despiking data outliers [59], double coordinate rotation [60], high-pass and low-pass filtering [56,61], and Webb-Pearman-Leuning (WPL) correction [62]. Fluxes were calculated at 30-minute block averages. Flux-footprint analysis’ were performed following the Kljun et al. [63] parametrization. Precipitation events were not explicitly removed from the dataset. However, diagnostic/error flags associate with IRGA and SAT instrument failure, including during heavy rainfall, were used as a filter. A requirement of 80% data coverage was applied for each 30-minute averaging interval.

#### 2.5.2. Flux Partitioning

Eddy covariance NEE measurements were partitioned into gross ecosystem productivity (GPP) (µmol m^−2^ s^−1^) and respiration (R_eco_) (µmol m^−2^ s^−1^). R_eco_ was calculated using the Reichstein et al. [64] partitioning algorithm. Periods of poor mixing, identified by low frictional velocity (u* < 0.21 m s^−1^) and representing possible unreliable NEE measurements, were removed. A light-response curve model [65,66,67,68] was used as an independent method for GPP derivation and calculations of apparent quantum yield. This method uses rectangular hyperbolic functions to express the response of photosynthesis to radiation, using the general expression:

(11) $$\;\mathrm{NEE}=-\left(\frac{A_{max}\cdot \alpha \cdot \mathrm{PPFD}}{A_{max}+\alpha \cdot \mathrm{PPFD}}\right)+R_{10}Q_{10}^{\left(T_{\mathrm{air}}-10\right)/10}\;$$

where *A_max_* is the maximum carbon assimilation, or GPP, (µmol m^−2^ s^−1^) at maximum photosynthetic photon flux density (PPFD) (µmol m^−2^ s^−1^); α is the apparent quantum yield calculated from the initial slope of the light-response curve (mol CO_2_ mol^−1^ PPFD); *R*_10_ is the ecosystem respiration rate at 10 °C (µmol m^−2^ s^−1^); *Q*_10_ is the respiration temperature response coefficient during temperature changes of 10 °C; and T_air_ is the air temperature (°C). *A_max_*, α, *R*_10_ and *Q*_10_ variables are resulting outputs of the non-linear least square, Gauss-Newton, regressions applied (using the statistical package Systat10, Systat Software, Inc., San Jose, CA, USA, 2000), of the input diurnal meteorological (PPFD and T_air_) and NEE flux data. Before light-response curves were derived, NEE data was binned over two days to derive representative diurnal patterns. 

## 3. Results

### 3.1. Sensor Calibrations and Validation

**Figure 2:**
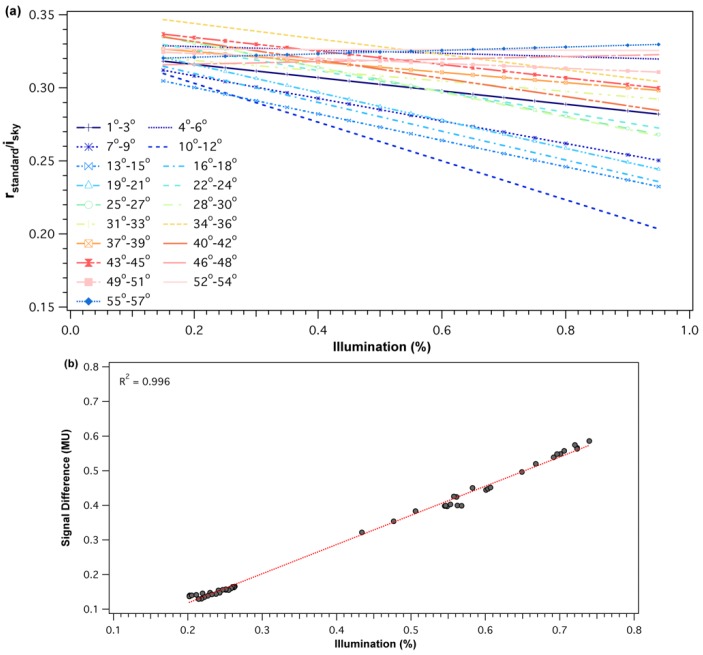
(**a**) Example of midday and diurnal cross-calibration functions for the 532 nm SRS-PRI fore-optic. Cross-calibration ratios are shown as a function of sun elevation (binned every 3°). Illumination of 100% represents clear and sunny sky conditions. (**b**) Example of the observed signal difference (radiance − irradiance), in machine units, as a function of illumination percentage for the 41°–43° solar elevation bin. Radiance and irradiance measurements were collected from the 532 nm SRS-PRI sensor pair.

**Figure 3:**
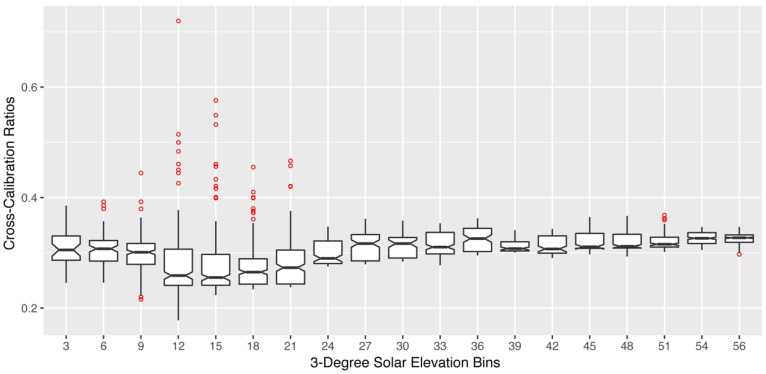
Boxplot distribution of diurnal cross-calibration ratios for the 532 nm SRS-PRI fore-optic. Each boxplot represents a 3° solar elevation bin. Red points represent outliers and were observed more often in low solar elevation bins.

For each of the SRS-PRI sensor pairs (532 nm and 570 nm), cross-calibration ratios showed a strong linear relationship to illumination but varied in response to solar elevation. At low elevations, functions had steeper slopes and, therefore, showed higher sensitivity to illumination changes (Figure 2a). Illumination sensitivity decreased as solar elevation increased and functions were near horizontal at solar elevations of >52°, which represented peak solar noon elevations at our site. For both the 532 nm and 570 nm pairs, the difference in signal (radiance − irradiance) between the hemispherical and field stop fore-optics over the white balance (Figure 2b) decreased with diffused light observed cloudy conditions. This effect leads to calibration functions with negative slopes where cross-calibration ratios were higher during low illumination than high illumination. Boxplot distributions of cross-calibration functions (Figure 3) show an overall decrease in variability as sun elevation increased towards higher solar elevations. This was expected as low sun angles cause higher specular reflectance. However, generally, cross-calibration ratios were within range of theoretical radiance/irradiance ratio value of 0.318 [69].

**Figure 4:**
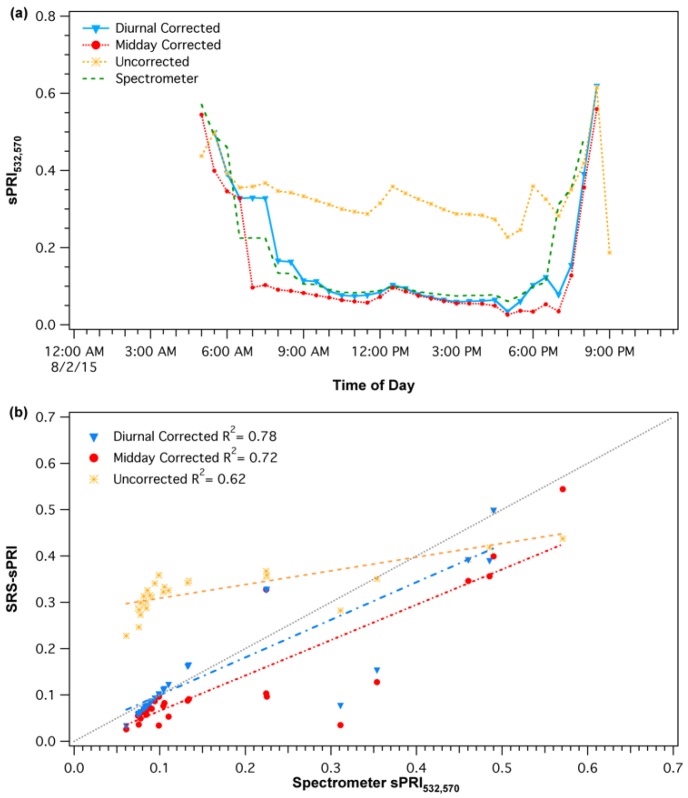
(**a**) Comparison of uncorrected and corrected (diurnal and midday) PRI diurnal patterns. (**b**) Comparison of spectrometer PRI measurements with uncorrected and corrected SRS-PRI measurements. The diurnal cross-calibration procedure shows the strongest and closest correlation to spectrometer readings and 1:1 (gray) line.

The diurnal corrections were applied by first matching the empirical cross-calibrations functions with the solar elevations of each SRS-PRI data points. Then, the derived percent illumination of each point was used to automatically determine the cross-calibration multipliers and derive new corrected reflectance SRS-PRI values. Scaled photochemical reflectance index (sPRI) values were derived from corrected SRS-PRI. A similar process was applied for the midday correction procedure; however, instead of accounting for continuous changes in solar elevation, only daily solar noon elevations were used to select the appropriate empirical cross-calibration response. Solar noon elevations at our site varied from 24.28° to 56.36° (fall and summer, respectively). Both diurnal and midday cross-calibration procedures (Figure 4a) had a significant effect on the sPRI diurnal pattern. Each of the corrections caused a downward shift in midday sPRI values as well as a sharper recovery during the start and end of the day. These patterns more closely represented the spectrometer derived sPRI diurnal pattern than that formed by the uncorrected SRS-sPRI values. In particular, the dynamic pattern of the diurnal cross-calibration best represented the reference spectrometer shape. Regression analysis (Figure 4b) of diurnal corrected SRS-sPRI values and spectrometer sPRI showed a strong linear correlation (*R*^2^ = 0.78, *p* < 0.05). Diurnally corrected and uncorrected values were also significantly correlated to spectrometer sPRI (*R*^2^ = 0.72, *p* < 0.05 and *R*^2^ = 0.62, respectively).

### 3.2. Meteorological and Carbon Flux Data

**Figure 5:**
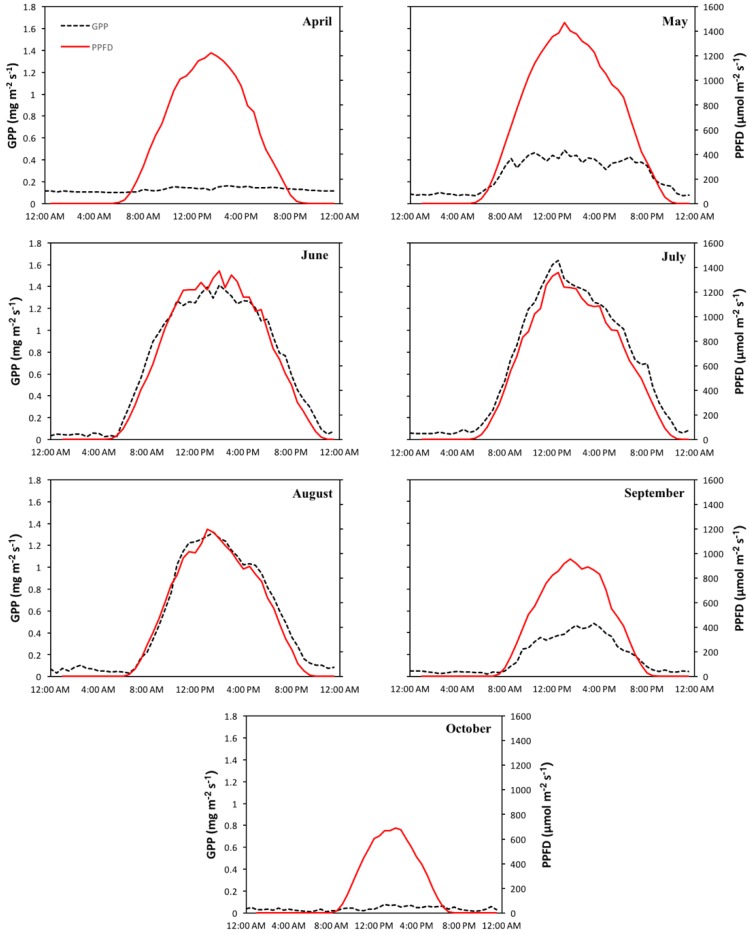
Mean diurnal monthly pattern of gross primary productivity (GPP) (black dotted line) and photosynthetic photon flux density (red line) for the growing season (April–October).

Seasonal time series of net ecosystem exchange (NEE), gross primary productivity (GPP), and respiration (R_eco_) were compared to meteorological variables and allowed the identification of climatic drivers directing intra-seasonal variability in carbon fluxes. Key connections between carbon fluxes and meteorological variables provided indications of the underlying mechanisms affecting vegetation efficiency over the season and during individual phenologic stages (maturity and leaf senescence). A more detailed analysis of carbon fluxes and meteorological data can be found in Appendix A. PPFD appeared to be a strong driver of productivity during maturity. Comparison of diurnal patterns of GPP and PPFD (Figure 5) showed significant correlations during the months of June (*R*^2^ = 0.91, *p* < 0.05), July (*R*^2^ = 0.90, *p* < 0.05), August (*R*^2^ = 0.85, *p* < 0.05), and September (*R*^2^ = 0.80, *p* < 0.05). Productivity during the leaf senescence months, April and October, showed no association to PPFD conditions. 

**Figure 6:**
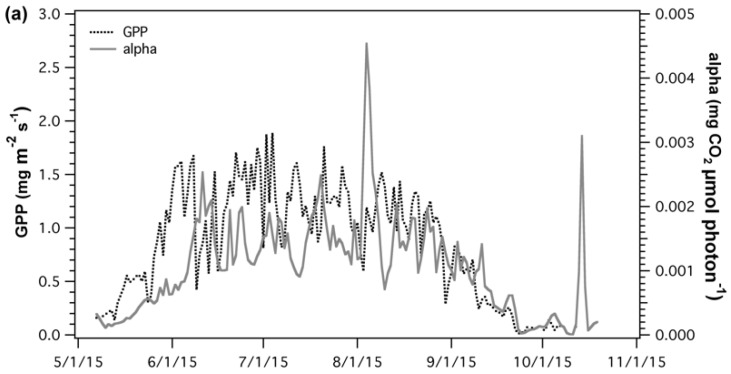

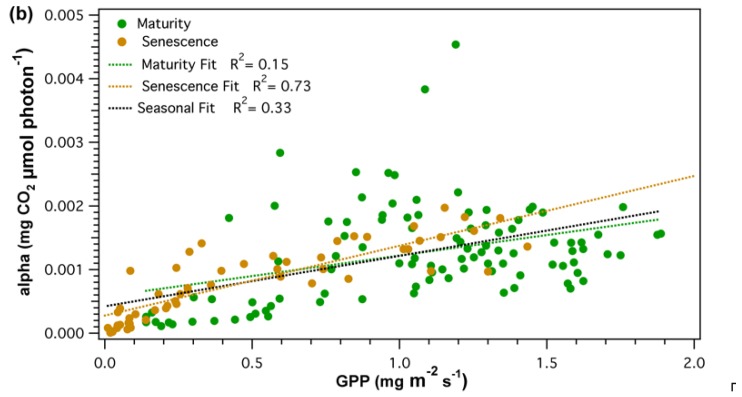
(**a**) Time series and (**b**) regression analysis of gross primary productivity (GPP) and apparent canopy quantum yield (alpha). Correlation between GPP and alpha was analyzed for the whole season (black), maturity (green), and leaf senescence (brown).

A combination of meteorological factors affecting apparent quantum yield (α) made it hard to determine the proportion of effect of each variable and how these may change over the growing season. However, some key components were identified and further discussed in the Appendix A. Seasonal comparison of GPP and α (Figure 6a) were poorly correlated (*R*^2^ = 0.33) over the whole season. However, during leaf senescence, (Figure 6b) as GPP starts to decline, GPP and α showed similar temporal changes and were significantly correlated (*R*^2^ = 0.73, *p* < 0.05).

### 3.3. Comparison of Light Use Efficiency Parameters and Canopy Structure Parameters

**Figure 7:**
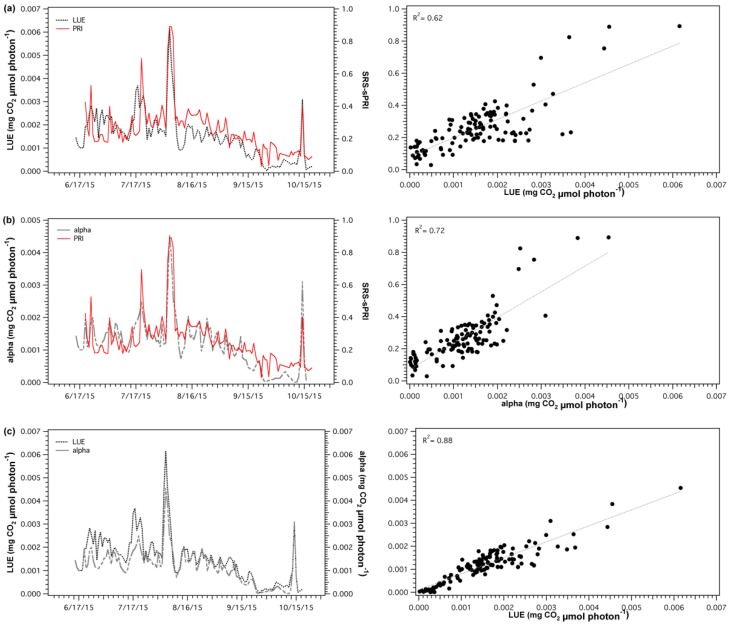
Time series (left panels) and regression plots (right panels) of (**a**) light use efficiency (LUE) and scaled photochemical reflectance index from SRS sensors (SRS-sPRI), (**b**) apparent quantum yield (alpha) and SRS-sPRI, and (**c**) LUE and α. Dotted lines in regressions plots (right panels) show linear regressions fitted to the data.

SRS-sPRI values were compared to LUE and α to determine its suitability as a proxy of light use efficiency. Comparison of SRS-sPRI and LUE time series (Figure 7a) showed similar seasonal patterns and were significantly correlated (*R*^2^ = 0.62, *p* < 0.05). PRI was also able to resolve periods of high LUE changes. 

**Figure 8:**
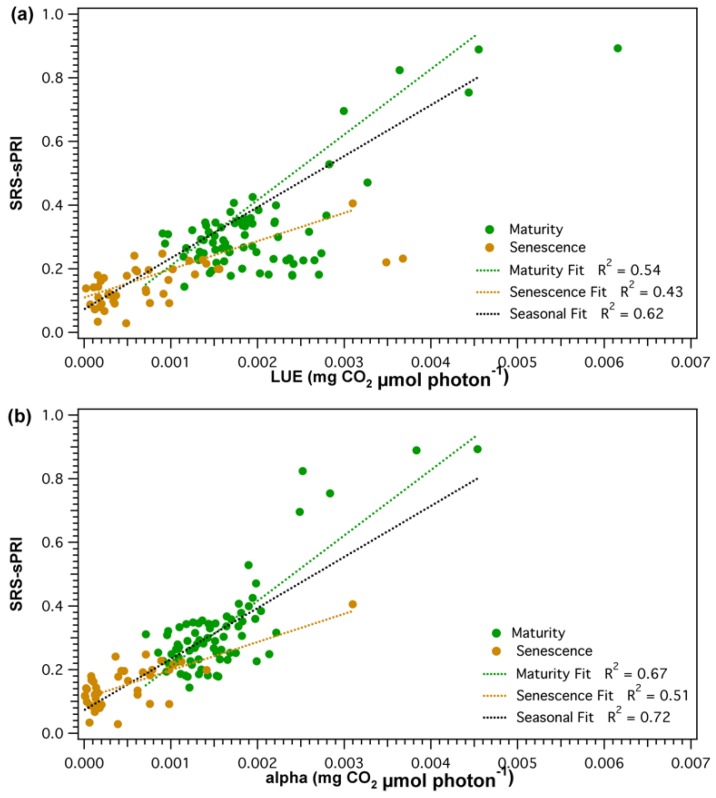
Seasonal (black), maturity (green), and leaf senescence (brown) correlations of scaled photochemical reflectance index from SRS sensors (SRS-sPRI) and (**a**) light use efficiency (LUE), and (**b**) apparent quantum yield (alpha). Dotted lines show linear fits.

However, smaller fluctuations observed throughout the LUE time series were not as clearly resolved by the SRS sensors. Further separation of the LUE–SRS-sPRI relationship by phenologic stages (Figure 8a) showed higher correlation during maturity (*R*^2^ = 0.54) than during leaf senescence (*R*^2^ = 0.43). Also, higher variability in SRS-sPRI values was observed during maturity (σ = 0.15) than during leaf senescence (σ = 0.069). Correlation analysis of SRS-sPRI and α (Figure 7b) showed a strong significant correlation (*R*^2^ = 0.72, *p* < 0.05). Temporal dynamics of α were also able to be better tracked by SRS-sPRI, especially during maturity (Figure 8b) (*R*^2^ = 0.67). During leaf senescence (Figure 8b), SRS-sPRI started to increasingly depart from α values leading to a weaker relationship (*R*^2^ = 0.51). Comparison of LUE and α (Figure 7c) resulted in highly correlated arrays (*R*^2^ = 0.88, *p* < 0.05). The α time series followed the dynamic changes in LUE but with less short term variations, likely due to the 2-day temporal binning methodology used to calculate the α term.

**Figure 9:**
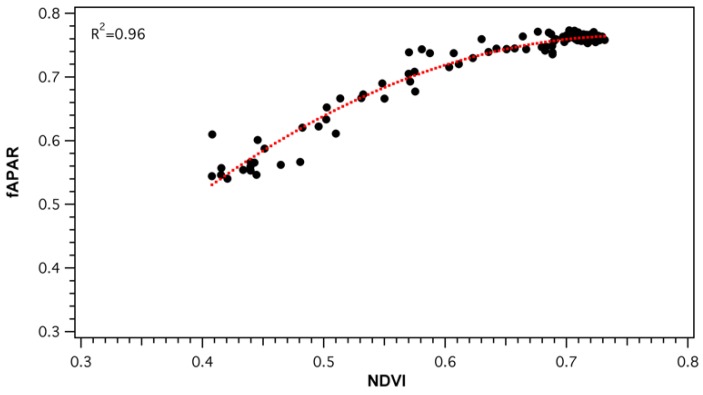
NDVI-*f*APAR relationships derived from daily values. NDVI was calculated from broadband spot radiometer sensors. *f*APAR was derived from the wireless sensor network (WSN) composing of 36 nodes measuring canopy transmitted light. Dotted line represents polynomial regression fitted to the data: y = −1.9596$\;\chi^{2}$ + 2.954x − 0.3484, *R*^2^ = 0.96.

**Figure 10:**
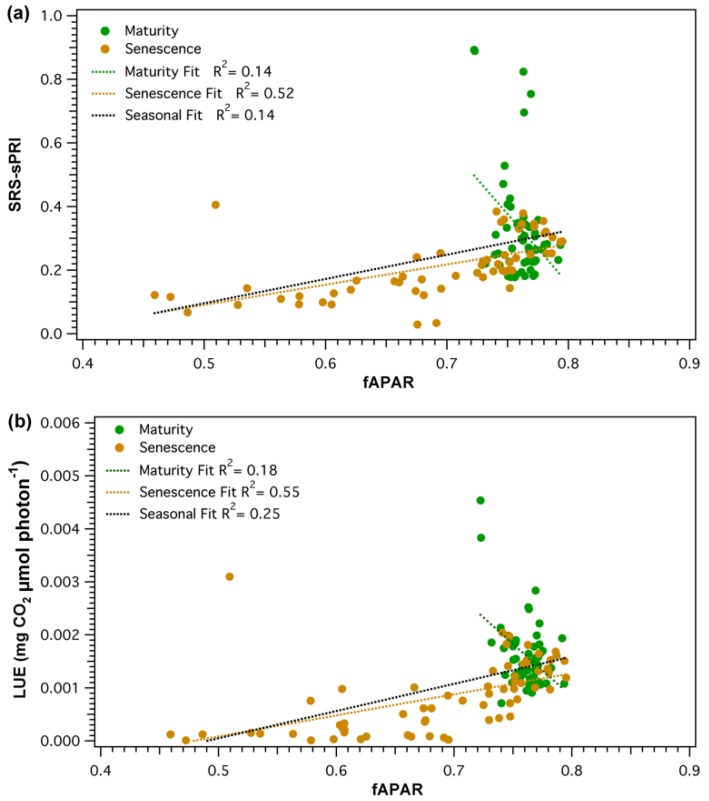
Relationship between fraction of absorbed photosynthetically active radiation (*f*APAR) and (**a**) scaled photochemical reflectance index from SRS sensors (SRS-sPRI), and (**b**) light use efficiency (LUE). Comparisons are shown for the combined season (black) as well separated into maturity (green) and leaf senescence (brown) phenologic stages.

Parametrization of light use efficiency must be done on the basis of green vegetation [5]. As collected fAPAR values represented the total light conditions of the forest stand, an NDVI-*f*APAR relationship (Figure 9) was established to ensure that fAPAR was, in fact, a good proxy of vegetation greenness changes. Daily NDVI and *f*APAR values strongly correlated (*R*^2^ = 0.96, *p* < 0.05), but the relationship was non-linear. NDVI was seen to saturate as values reached above approximately 0.65 (*f*APAR ≈ 0.75). The effect of canopy structure and pigment pool changes on the PRI signal was evaluated by comparing SRS-sPRI values with measured *f*APAR. Analysis of the seasonal relationship (Figure 10a) showed *f*APAR and SRS-sPRI as being poorly correlated (*R*^2^ = 0.14, *p* > 0.05). The seasonal time series was further divided into maturity and leaf senescence, and correlations were derived (Figure 10a). During maturity, we observed a poor relationship between *f*APAR and SRS-sPRI values, suggesting a minimal effect of canopy structure on PRI signals. However, during leaf senescence, a linear relationship (*R*^2^ = 0.52, *p* < 0.05) formed between *f*APAR and SRS-sPRI values. To confirm the effect of canopy structure on efficiency during maturity and leaf senescence, the same comparisons were performed using LUE values, and lead to comparable results (Figure 10b).

### 3.4. Light Use Efficiency Models

**Figure 11:**
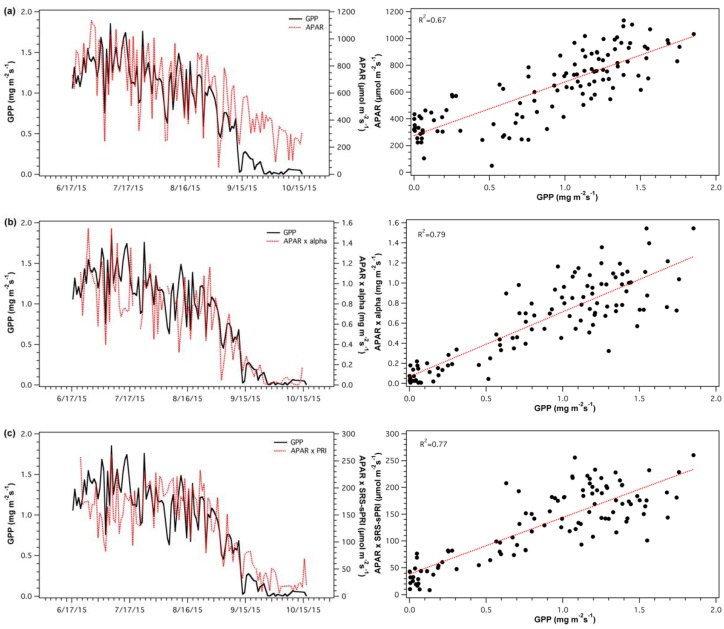
Time series (top panels) and regressions (bottom panels) of (**a**) absorbed photosynthetically active radiation (APAR) and gross primary productivity (GPP), (**b**) light use efficiency (LUE) model driven by apparent quantum yield (α) and GPP, and (**c**) light use efficiency (LUE) model driven by scaled photochemical reflectance index from SRS sensors (sPRI) and GPP.

SRS-sPRI and α values were used to derive light use efficiency models and compared to GPP to assess each model’s accuracy. APAR values were also compared to GPP (Figure 11a) to determine its contributing proportion to the overall model. APAR closely tracked temporal changes in GPP throughout maturity, however, during leaf fall GPP and APAR show differing paths. The LUE model constructed from α values (Figure 11b) was significantly correlated to GPP (*R*^2^ = 0.79, *p* < 0.05). The model’s closest association to GPP occurred during leaf senescence, as GPP started to decline. The light use efficiency model driven by the SRS-sPRI dataset (Figure 11c) had very similar results to that of the α based model and showed significant correlations to GPP (*R*^2^ = 0.77, *p* < 0.05). Again, the GPP and models’ paths were most closely associated during senescence, as leaf fall initiated. 

## 4. Discussion

As suggested in other studies [70,71,72,73], our results show that a cross-calibration must be performed to properly calibrate automatic optical sensors. Uncalibrated SRS-PRI values did not have good agreement with spectrometer PRI values. Unlike results from Gamon et al. [44], both diurnal and midday calibrations noticeably improved the ability to resolve diurnal PRI patterns. Although we do not know the reason for this difference in calibration outcomes, one possibility may be that our aspen stand could represent more a heterogeneous structure and illumination conditions. Wind conditions that are common at our calibration site could help randomize the leaf orientation so that both the spectrometer and SRS sensors measure similar sunlit and shaded leaf proportions. Our contrasting results advocate for the importance of characterizing site-specific light fields that can vary in complexity between different canopy structures and may affect the ability to resolve diurnal dynamics accurately. A lack of ability to accurately resolve diurnal PRI patterns becomes problematic when diurnal patterns of the xanthophyll cycle and light use efficiency is wanted [39,44,74]. 

Although cross-calibrations provided more accurate PRI values, errors remain. In general, the main discrepancy between the SRS-PRI sensors and spectrometer measurements occurred as we moved away from midday. This suggests that the factory SRS sensors sensitivity cannot resolve the full range of reflectance changes. Uncalibrated SRS sensors result in an overestimation of PRI and LUE and modeled GPP values if used within a LUE model. To prevent erroneous PRI values that can lead to the mischaracterization of light use efficiency, we recommend regular calibrations and corrections of SRS sensors [71,73]. Diurnal cross-calibration procedures confirmed that an offline calibration is possible and can be used to correct the effects of illumination and solar elevation on radiance and irradiance measurements. However, it is important to note that our procedure did not account for changes in solar azimuth that lead to changes in directional reflectance [75,76]. Solar azimuth changes will have a meaningful impact in high latitude ecosystems as angles will vary significantly from start to peak growing season. Real-time calibrations would account from changes in solar azimuth. However, given the constant manual intervention needed during real-time cross-calibrations, we believe our procedure to be a good compromise. The offline diurnal calibration was able to resolve diurnal PRI pattern (and xanthophyll cycle changes) more accurately than midday calibrations. The diurnal cross-calibrations results corroborate previous findings showing the effect of sun-canopy-sensor geometry on PRI values [33,43,77,78]. 

The strong correlation between SRS-sPRI and LUE (*R*^2^ = 0.62) show that automated SRS-PRI sensors can be used as a proxy for light use efficiency. It is important to note the observed relationship between SRS-sPRI and LUE was stronger than the mean of 27 broadleaf forests reported correlations (*R*^2^ = 0.59) reviewed by Zhang et al. [24]. Comparison between SRS-sPRI and apparent canopy quantum yield showed an even stronger correlation with an *R*^2^ = 0.72 that represents the upper third quartile boundary of the broadleaf forest PRI-LUE correlation meta-analysis. Although quantum yield is not equivalent to light use efficiency, the strong correlation between α and LUE (*R*^2^ = 0.88) show that α can be used as an adequate proxy of LUE when PPFD is the primary driving variable of productivity. As such, the strong correlation of between SRS-sPRI and α provide even more evidence that PRI values from the SRS sensors can serve as a proxy for efficiency.

Although sPRI was well correlated to light use efficiency parameters, their association appeared to vary within different developmental stages. The close correlation between sPRI, LUE (*R*^2^ = 0.54) and α (*R*^2^ = 0.67) during maturity show ability to track the facultative changes of xanthophyll cycle pigment pools. This was further supported by the weak correlation between *f*APAR and sPRI (*R*^2^ = 0.14), indicating the low effect of vegetation structure that drives constitutive pigment pool changes [38]. During leaf senescence sPRI was less strongly correlated to efficiency parameters (light use efficiency *R*^2^ = 0.43, quantum yield *R*^2^ = 0.51) and correlated with *f*APAR (*R*^2^ = 0.52), suggesting that the PRI signal is influenced by canopy changes. In deciduous vegetation, senescence marks a period of substantial changes in canopy structure composition as leaves start to senesce and fall. During this marked period of structural variation, senescence PRI would most likely represent a mixture of facultative and constitutive changes, with constitutive changes confounding the physiological signal from leaves. This aligns with studies [79,80] suggesting that dominant effects of canopy structure can confound physiological leaf traits detection. Future parametrization of LUE models will need to take into account PRI’s contrasting effect on photosynthetic activity during different time periods [21]. The high temporal resolution of the automatic SRS-PRI sensors and their ability to serve as a proxy of LUE at different temporal scales could be useful towards the better parametrization of LUE models. 

A close analysis of PRI and light use efficiency parameters provided some underlying mechanism affecting efficiency at our site. The close relationship of α and GPP during senescence indicate that efficiency changes are influenced by canopy structure, this being the primary driving mechanism of GPP changes. Coinciding efficiency peaks present in sPRI, LUE and α time series were associated with high water availability (soil and atmospheric), lower temperatures and low PPFD. In the context of PRI, we can infer that periods of low PPFD would account for the little need for non-photochemical quenching as canopy would not be exposed to saturating light conditions. Diffused light conditions have been shown to result in higher canopy light use efficiency [81,82,83] and could explain the increases in LUE rates. This effect of diffused light on light use efficiency rates has been difficult to account for in remote sensing modeling as most of the driving data come from non-continuous satellite-based imagery. Continuous PRI measurements have the potential to solve this, as illumination can be used to classify continuous datasets into illumination groups and used to empirically describe the effects of different light conditions on LUE through time.

Many described canopy scale variables have been described that can confound PRI signals including canopy structure, view and illumination angles, soil types, shadow fractions, phenology and pigment pools [19,21,38,84,85]. One approach to mitigate some of these effects is through the normalization of PRI (calculating daily ∆PRI) [86]. This has been shown to reduce the effects of canopy structure, vegetation cover, and soil background. Although not applied in this study, PRI normalization is a topic of future work as it may improve PRI-LUE relationships from continuous sensors. Some of the variables previously mentioned also impact our sPRI dataset. We can also assume that our continuous measurements will have a wide range of impacts as the higher temporal resolution will naturally capture more of these interactions. Although quantifying the individual effect of each impacting variable is difficult, we believe that continuous datasets provide the ability to explicitly examine the cause of changing PRI over time and space. This has been a criticism of studies using non-continuous PRI measurements such as those from satellite platforms, where their low temporal resolution cannot resolve short-term reflectance changes and instead changes primarily represent changing pigment pools [38,39].

Both the sPRI and α LUE models were able to track the overall progression of GPP. Still, some of the short-term dynamic changes observed in GPP during maturity were better captured by APAR the sPRI and α derived LUE models. We can attribute this to the strong association between incident light and productivity during peak GPP months. During peak GPP months, canopy structure remains mostly unchanged; thus APAR variability is driven by PPFD dynamics. During leaf senescence, PPFD was no longer associated with GPP and lead to the differences seen between APAR and GPP trends. Overall, the α driven LUE model showed to be the most accurate. The sPRI LUE model followed closely and was able to track seasonal changes in productivity. Evaluation of APAR, sPRI and LUE model results indicate that construction of LUE models needs to consider the changing contribution of the individual model variables through time and within different ecosystems. This follows Garbulsky et al. [14] concept suggesting that canopy efficiency can change between vegetation and environmental conditions. Exploration of this concept has been hard to test due to lack of continuous datasets that would allow mechanistic and comparative analysis within and across biomes. Continuous SRS-sPRI offer new opportunities to explore diurnal and seasonal changes in vegetation physiology.

## 5. Conclusions

An offline diurnal calibration was able to characterize the optical sensor’s response and allowed us to resolve diurnal and seasonal PRI patterns. Corrected PRI values from SRS sensor proved to be appropriate proxies of light use efficiency. Our results contribute to the growing research indicating that continuous PRI sensors can be used to track diurnal and seasonal changes in efficiency. Still, it is important to define facultative and constitutive proportions within the PRI observations and how this may change over different ecosystems and growth stages. Studies on the explicit effect of environmental conditions on PRI signals will also prove to be important since the higher resolution of continuous measurements will need to be accurately characterized. In general, we see continuous PRI sensors as having numerous benefits for explicitly examining the cause of changing PRI, LUE, and productivity over time and space. For this reason, we see this technology of great value for the flux, remote sensing and modeling community.

## Figures and Tables

**Figure A1:**
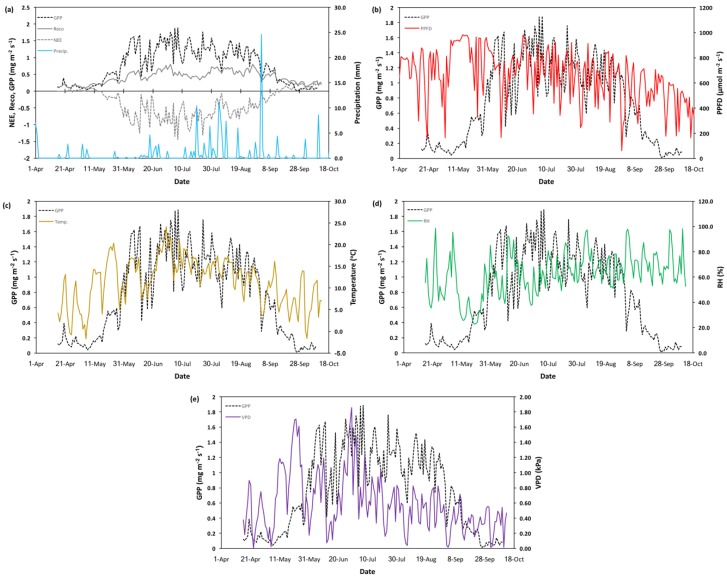
Gross primary productivity (GPP) and seasonal meteorological parameters for the 2015 growing season at the Peace River Environmental Monitoring Super Site. From top to bottom: (**a**) daily average net ecosystem exchange and ecosystem respiration, (**b**) photosynthetic photon flux density (PPFD), (**c**) temperature, (**d**) relative humidity (RH), and (**e**) vapor pressure deficit (VPD).

**Figure A2:**
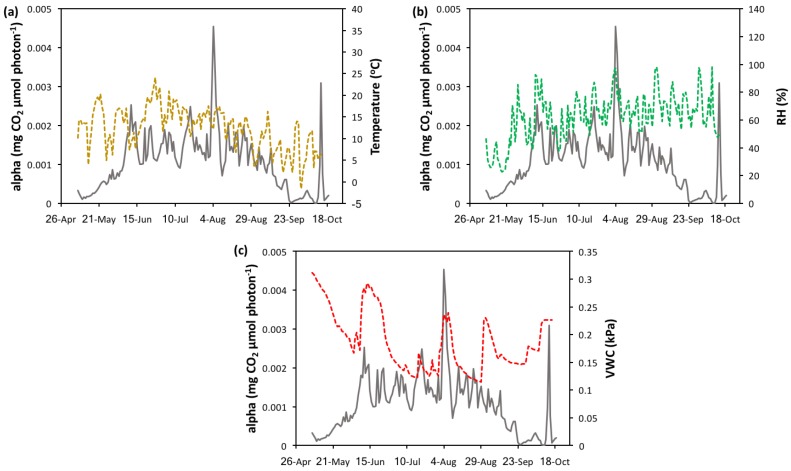
Time series of apparent quantum yield and (**a**) temperature, (**b**) relative humidity (RH), and (**c**) soil volumetric water moisture (VWC). Apparent quantum yield values were derived from eddy covariance measurements.

## Appendix A

The seasonal time series of NEE, GPP, R_eco_, and key meteorological variables are shown in Figure A1. Seasonal patterns of NEE (Figure A1a) are similar to those usually observed in northern deciduous forests, resulting from the known phenologic cycle. During spring and fall dormancy, NEE values are in the positive range, suggesting a higher proportion of respiration than photosynthesis that leads to a net carbon source. As leaf flush occurred, the opposite happens with productivity outpacing respiration, leading to increasingly negative NEE and net carbon sequestration. NEE and GPP progressively increased throughout the season (Figure A1a), reaching peak productivity during July (1.4 ± 0.02 mg m^−2^ s^−1^ and 1.8 ± 0.03 mg m^−2^ s^−1^, respectively). Peak respiration (0.80 ± 0.03 mg m^−2^ s^−1^) also occurred during July but slightly offset from peak productivity. NEE, GPP, and R_eco_ showed a gradual decline into leaf senescence. The length of the growing season was approximately 170 days (± 7 days), spanning from 15 April to 1 October, 2015.

Seasonal trends in temperature (Figure A1c) show a continuous increase from the beginning of the season to late June, after which temperature progressively decreases until the end of the season (October). Overall, relative humidity (Figure A1d) increases until mid-June and then remains stable during the remainder of the growing season. VPD seasonal changes (Figure A1e) showed an initial increase during the start of the season, followed by a sharp decrease during mid-June and then remaining low and stable from July until end of the season. Beyond these general seasonal changes, short-term changes in climatic status directed intra-seasonal variability in productivity. Throughout the season productivity was linked to air temperature (Figure A1c), particularly during spring and fall warming/cooling events, where warming was associated with increased GPP and cooling with seasonal decreases. The largest drops in GPP and R_eco_ values occurred during periods were temperature reached approached <5 °C. VPD (Figure A1e) was principally influenced by temperature and shared similar patterns and associations to GPP. PPFD appeared to be the principal driver of productivity throughout large parts of the season (Figure A1b and Figure 5).

A combination of factors appears to affect apparent quantum yield (α) over the course of the growing season. Periods of low quantum yield occurred during high temperatures (Figure A2a), which we would expect from a heat-stressed ecosystem. The opposite relationship is observed when comparing RH and α (Figure A2b). In general, high α values coincide with periods of high relative humidity, which represent low evaporative demand. Peak α values correspond closely with soil water moisture (Figure A2b). An exception to this connection was observed during 31 August when a soil moisture peak is observed but α seems to decrease slightly. This decrease in α is associated with low PPFD (61.49 µmol m^−2^ s^−1^) and T_air_ (3.7 °C), indicating that these variables are limiting during this period.

## References

[1] Monteith J.L. (1972). Solar radiation and productivity in tropical ecosystems. J. Appl. Ecol..

[2] Monteith J.L. (1977). Climate and the efficiency of crop production in Britain. Philos. Trans. R. Soc. Lond. B.

[3] Huete A., Ponce-Campos G., Zhang Y., Restrepo-Coupe N., Ma X., Moran M. (2015). Monitoring photosynthesis from space. Land Resources Monitoring, Modeling, and Mapping with Remote Sensing.

[4] Schimel D., Pavlick R., Fisher J.B., Asner G.P., Saatchi S., Townsend P., Miller C., Frankenberg C., Hibbard K., Cox P. (2015). Observing terrestrial ecosystems and the carbon cycle from space. Glob. Chang. Biol..

[5] Gitelson A.A., Gamon J.A. (2015). The need for a common basis for defining light-use efficiency: Implications for productivity estimation. Remote Sens. Environ..

[6] Sellers P.J. (1985). Canopy reflectance, photosynthesis and transpiration. Int. J. Remote Sens..

[7] Sellers P.J. (1987). Canopy reflectance, photosynthesis, and transpiration, II. The role of biophysics in the linearity of their interdependence. Remote Sens. Environ..

[8] Myneni R.B., Williams D.L. (1994). On the relationship between FAPAR and NDVI. Remote Sens. Environ..

[9] Asrar G.M., Myneni R.B., Choudhury B.J. (1992). Choudhury. Spatial heterogeneity in vegetation canopies and remote sensing of absorbed photosynthetically active radiation: A modeling study. Remote Sens. Environ..

[10] Gobron N., Pinty B., Aussedat O., Chen J.M., Cohen W.B., Fensholt R., Gond V., Huemmrich K.F., Lavergne T., Mélin F. (2006). Evaluation of fraction of absorbed photosynthetically active radiation products for different canopy radiation transfer regimes: Methodology and results using Joint Research Center products derived from SeaWiFS against ground-based estimations. J. Geophys. Res. Atmos..

[11] Widlowski J.-L. (2010). On the bias of instantaneous FAPAR estimates in open-canopy forests. Agric. For. Meteorol..

[12] Field C.B., Gamon J.A., Peñuelas J., Schulze E.-D., Caldwell M.M. (1994). Remote sensing of photosynthesis. Ecophysiology of Photosynthesis.

[13] Garbulsky M.F., Peñuelas J., Papale D., Ardö J., Goulden M.L., Kiely G., Richardson A.D., Rotenberg E., Veenendaal E.M., Filella I. (2010). Patterns and controls of the variability of radiation use efficiency and primary productivity across terrestrial ecosystems. Glob. Ecol. Biogeogr..

[14] Garbulsky M.F., Peñuelas J., Gamon J., Inoue Y., Filella I. (2011). The photochemical reflectance index (PRI) and the remote sensing of leaf, canopy and ecosystem radiation use efficiencies: A review and meta-analysis. Remote Sens. Environ..

[15] Jarvis P.G., Leverenz J.W. (1983). Productivity of temperate, deciduous and evergreen forests. Physiological plant ecology IV.

[16] Ruimy A.L., Jarvis P.G., Baldocchi D.D., Saugier B. (1995). CO_2_ fluxes over plant canopies and solar radiation: A review. Adv. Ecol. Res..

[17] Gamon J.A., Serrano L., Surfus J. (1997). The photochemical reflectance index: An optical indicator of photosynthetic radiation use efficiency across species, functional types, and nutrient levels. Oecologia.

[18] Ahl D.E., Gower S.T., Mackay D.S., Burrows S.N., Norman J.M., Diak G.R. (2004). Heterogeneity of light use efficiency in a northern Wisconsin forest: Implications for modeling net primary production with remote sensing. Remote Sens. Environ..

[19] Sims D.A., Gamon J.A. (2002). Relationships between leaf pigment content and spectral reflectance across a wide range of species, leaf structures and developmental stages. Remote Sens. Environ..

[20] Jenkins J.P., Richardson A.D., Braswell B.H., Ollinger S.V., Hollinger D.Y., Smith M.L. (2007). Refining light-use efficiency calculations for a deciduous forest canopy using simultaneous tower-based carbon flux and radiometric measurements. Agric. For. Meteorol..

[21] Gamon J.A., Huemmrich K.F., Wong C.Y., Ensminger I., Garrity S., Hollinger D.Y., Noormets A., Peñuelas J. (2016). A remotely sensed pigment index reveals photosynthetic phenology in evergreen conifers. Proc. Natl. Acad. Sci. USA.

[22] Zhang C., Filella I., Liu D., Ogaya R., Llusià J., Asensio D., Peñuelas J. (2017). Photochemical reflectance index (PRI) for detecting responses of diurnal and seasonal photosynthetic activity to experimental drought and warming in a mediterranean shrubland. Remote Sens..

[23] Peñuelas J., Filella I., Gamon J.A. (1995). Assessment of photosynthetic radiation-use efficiency with spectral reflectance. New Phytol..

[24] Zhang C., Filella I., Garbulsky M., Peñuelas J. (2016). Affecting factors and recent improvements of the photochemical reflectance index (PRI) for remotely sensing foliar, canopy and ecosystemic radiation-use efficiencies. Remote Sens..

[25] Garbulsky M.F., Filella I., Verger A., Peñuelas J. (2014). Photosynthetic light use efficiency from satellite sensors: From global to Mediterranean vegetation. Environ. Exp. Bot..

[26] Gamon J.A., Field C.B., Bilger W., Björkman O., Fredeen A.L., Peñuelas J. (1990). Remote sensing of the xanthophyll cycle and chlorophyll fluorescence in sunflower leaves and canopies. Oecologia.

[27] Gamon J.A., Penuelas J., Field C.B. (1992). A narrow-waveband spectral index that tracks diurnal changes in photosynthetic efficiency. Remote Sens. Environ..

[28] Björkman O., Demmig-Adams B. (1995). Regulation of photosynthetic light energy capture, conversion, and dissipation in leaves of higher plants. Ecophysiology of Photosynthesis.

[29] Demmig-Adams B., Adams W.W., Logan B.A., Verhoeven A.S. (1995). Xanthophyll cycle-dependent energy dissipation and flexible photosystem II efficiency in plants acclimated to light stress. Funct. Plant Biol..

[30] Demmig-Adams B., Adams W.W. (1996). The role of xanthophyll cycle carotenoids in the protection of photosynthesis. Trends Plant Sci..

[31] Gamon J.A. (1993). The Dynamic 531-Nanometer A Reflectance Signal: A Survey of Twenty Angiosperm Species, Gilmore Hall 202-3050 Maile Way Honolulu, Hawaii 96822 Office of the Director.

[32] Gamon J.A., Surfus J.S. (1999). Assessing leaf pigment content and activity with a reflectometer. New Phytol..

[33] Barton C.V., North P.R. (2001). Remote sensing of canopy light use efficiency using the photochemical reflectance index: Model and sensitivity analysis. Remote Sens. Environ..

[34] Cheng Y.B., Middleton E.M., Zhang Q., Corp L.A., Dandois J., Kustas W.P. (2012). The photochemical reflectance index from directional cornfield reflectances: Observations and simulations. Remote Sens. Environ..

[35] Stylinski C., Gamon J., Oechel W. (2002). Seasonal patterns of reflectance indices, carotenoid pigments and photosynthesis of evergreen chaparral species. Oecologia.

[36] Filella I., Porcar-Castell A., Munné-Bosch S., Bäck J., Garbulsky M.F., Peñuelas J. (2009). PRI assessment of long-term changes in carotenoids/chlorophyll ratio and short-term changes in de-epoxidation state of the xanthophyll cycle. Int. J. Remote Sens..

[37] Garrity S.R., Eitel J.U., Vierling L.A. (2011). Disentangling the relationships between plant pigments and the photochemical reflectance index reveals a new approach for remote estimation of carotenoid content. Remote Sens. Environ..

[38] Gamon J.A., Berry J.A. (2012). Facultative and constitutive pigment effects on the Photochemical Reflectance Index (PRI) in sun and shade conifer needles. Isr. J. Plant Sci..

[39] Gitelson A.A., Gamon J.A., Solovchenko A. (2017). Multiple drivers of seasonal change in PRI: Implications for photosynthesis 2. Stand level. Remote Sens. Environ..

[40] Nichol C.J., Lloyd J.O., Shibistova O., Arneth A., Röser C., Knohl A., Matsubara S., Grace J. (2002). Remote sensing of photosynthetic-light-use efficiency of a Siberian boreal forest. Tellus B Chem. Phys. Meteorol..

[41] Goerner A., Reichstein M., Tomelleri E., Hanan N., Rambal S., Papale D., Dragoni D., Schmullius C. (2011). Remote sensing of ecosystem light use efficiency with MODIS-based PRI. Biogeosciences.

[42] Gamon J.A. (2015). Reviews and syntheses: Optical sampling of the flux tower footprint. Biogeosciences.

[43] Garrity S.R., Vierling L.A., Bickford K. (2010). A simple filtered photodiode instrument for continuous measurement of narrowband NDVI and PRI over vegetated canopies. Agric. For. Meteorol..

[44] Gamon J.A., Kovalchuck O., Wong C.Y., Harris A., Garrity S.R. (2015). Monitoring seasonal and diurnal changes in photosynthetic pigments with automated PRI and NDVI sensors. Biogeosciences.

[45] Van Leeuwen M., Kremens R.L., van Aardt J. (2015). Tracking diurnal variation in photosynthetic down-regulation using low cost spectroscopic instrumentation. Sensors.

[46] Alberta Environmental Protection (1994). Natural Regions and Subregions of Alberta: A Summary, Publ. I/531 and Map, 1 Sheet.

[47] Atmospheric Environment Service (1982). Canadian Climate Normals (19511980).

[48] Soil Classification Working Group (1998). The Canadian System of Soil Classification.

[49] Rahman A.F., Cordova V.D., Gamon J.A., Schmid H.P., Sims D.A. (2004). Potential of MODIS ocean bands for estimating CO_2_ flux from terrestrial vegetation: A novel approach. Geophys. Res. Lett..

[50] Goerner A., Reichstein M., Rambal S. (2009). Tracking seasonal drought effects on ecosystem light use efficiency with satellite-based PRI in a Mediterranean forest. Remote Sens. Environ..

[51] Gamon J.A., Rahman A.F., Dungan J.L., Schildhauer M., Huemmrich K.F. (2006). Spectral Network (SpecNet)—What is it and why do we need it?. Remote Sens. Environ..

[52] Ryan P.J., Stolzenbach K.D., Harleman D.R.F. (1972). Engineering Aspects of Heat Disposal from Power Generation.

[53] Rankine C.J., Sanchez-Azofeifa G.A., MacGregor M.H. Seasonal wireless sensor network link performance in boreal forest phenology monitoring. Proceedings of the 2014 Eleventh Annual IEEE International Conference on Sensing, Communication, and Networking (SECON).

[54] Huemmrich K.F., Black T.A., Jarvis P.G., McCaughey J.H., Hall F.G. (1999). High temporal resolution NDVI phenology from micrometeorological radiation sensors. J. Geophys. Res. Atmos..

[55] Baldocchi D.D., Hincks B.B., Meyers T.P. (1988). Measuring biosphere-atmosphere exchanges of biologically related gases with micrometeorological methods. Ecology.

[56] Moncrieff J.B., Massheder J.M., De Bruin H., Elbers J., Friborg T., Heusinkveld B., Kabat P., Scott S., Søgaard H., Verhoef A. (1997). A system to measure surface fluxes of momentum, sensible heat, water vapour and carbon dioxide. J. Hydrol..

[57] Aubinet M., Grelle A., Ibrom A., Rannik Ü., Moncrieff J., Foken T., Kowalski A.S., Martin P.H., Berbigier P., Bernhofer C. (1999). Estimates of the annual net carbon and water exchange of forests: The EUROFLUX methodology. Advances in Ecological Research.

[58] Moore C.J. (1986). Frequency response corrections for eddy correlation systems. Bound.-Layer Meteorol..

[59] Vickers D., Mahrt L. (1997). Quality control and flux sampling problems for tower and aircraft data. J. Atmos. Ocean. Technol..

[60] Finnigan J.J. (2004). A re-evaluation of long-term flux measurement techniques part II: Coordinate systems. Bound.-Layer Meteorol..

[61] Moncrieff J., Clement R., Finnigan J., Meyers T. (2004). Averaging, detrending, and filtering of eddy covariance time series. Handbook of Micrometeorology.

[62] Webb E.K., Pearman G.I., Leuning R. (1980). Correction of flux measurements for density effects due to heat and water vapour transfer. Q. J. R. Meteorol. Soc..

[63] Kljun N., Calanca P., Rotach M.W., Schmid H.P. (2004). A simple parameterisation for flux footprint predictions. Bound.-Layer Meteorol..

[64] Reichstein M., Falge E., Baldocchi D., Papale D., Aubinet M., Berbigier P., Bernhofer C., Buchmann N., Gilmanov T., Granier A. (2005). On the separation of net ecosystem exchange into assimilation and ecosystem respiration: Review and improved algorithm. Glob. Chang. Biol..

[65] Flanagan L.B., Johnson B.G. (2005). Interacting effects of temperature, soil moisture and plant biomass production on ecosystem respiration in a northern temperate grassland. Agric. For. Meteorol..

[66] Glenn A.J., Flanagan L.B., Syed K.H., Carlson P.J. (2006). Comparison of net ecosystem CO_2_ exchange in two peatlands in western Canada with contrasting dominant vegetation, Sphagnum and Carex. Agric. For. Meteorol..

[67] Syed K.H., Flanagan L.B., Carlson P.J., Glenn A.J., Van Gaalen K.E. (2006). Environmental control of net ecosystem CO_2_ exchange in a treed, moderately rich fen in northern Alberta. Agric. For. Meteorol..

[68] Adkinson A.C., Syed K.H., Flanagan L.B. (2011). Contrasting responses of growing season ecosystem CO_2_ exchange to variation in temperature and water table depth in two peatlands in northern Alberta, Canada. J. Geophys. Res. Biogeosci..

[69] Monteith J.L. (1973). Principles of Environmental Physics.

[70] Balzarolo M., Anderson K., Nichol C., Rossini M., Vescovo L., Arriga N., Wohlfahrt G., Calvet J.C., Carrara A., Cerasoli S. (2011). Ground-based optical measurements at European flux sites: A review of methods, instruments and current controversies. Sensors.

[71] Harris A., Gamon J.A., Pastorello G.Z., Wong C.Y.S. (2014). Retrieval of the photochemical reflectance index for assessing xanthophyll cycle activity: A comparison of near-surface optical sensors. Biogeosciences.

[72] Drolet G., Wade T., Nichol C.J., MacLellan C., Levula J., Porcar-Castell A., Nikinmaa E., Vesala T. (2014). A temperature-controlled spectrometer system for continuous and unattended measurements of canopy spectral radiance and reflectance. Int. J. Remote Sens..

[73] Pacheco-Labrador J., Martín M.P. (2014). Nonlinear response in a field portable spectroradiometer: Characterization and effects on output reflectance. IEEE Trans. Geosci. Remote Sens..

[74] Gitelson A.A., Gamon J.A., Solovchenko A. (2017). Multiple drivers of seasonal change in PRI: Implications for photosynthesis 1. Leaf level. Remote Sens. Environ..

[75] Deering D.W., Leone P. (1986). A sphere-scanning radiometer for rapid directional measurements of sky and ground radiance. Remote Sens. Environ..

[76] Kimes D.S. (1983). Dynamics of directional reflectance factor distributions for vegetation canopies. Appl. Opt..

[77] Hilker T., Coops N.C., Hall F.G., Black T.A., Wulder M.A., Nesic Z., Krishnan P. (2008). Separating physiologically and directionally induced changes in PRI using BRDF models. Remote Sens. Environ..

[78] Galvão L.S., Breunig F.M., dos Santos J.R., de Moura Y.M. (2013). View-illumination effects on hyperspectral vegetation indices in the Amazonian tropical forest. Int. J. Appl. Earth Obs. Geoinf..

[79] Knyazikhin Y., Schull M.A., Stenberg P., Mottus M., Rautiainen M., Yang Y., Marshak A., Latorre Carmona P., Kaufmann R.K., Lewis P. (2013). Hyperspectral remote sensing of foliar nitrogen content. Proc. Natl. Acad. Sci. USA.

[80] Townsend P.A., Serbin S.P., Kruger E.L., Gamon J.A. (2013). Disentangling the contribution of biological and physical properties of leaves and canopies in imaging spectroscopy data. Proc. Natl. Acad. Sci. USA.

[81] Norman J.M., Arkebauer T.J. (1991). Predicting canopy light-use efficiency from leaf characteristics. Model. Plant Soil Syst..

[82] Choudhury B.J. (2001). Estimating gross photosynthesis using satellite and ancillary data: Approach and preliminary results. Remote Sens. Environ..

[83] Gu L., Baldocchi D., Verma S.B., Black T.A., Vesala T., Falge E.M., Dowty P.R. (2002). Advantages of diffuse radiation for terrestrial ecosystem productivity. J. Geophys. Res. Atmos..

[84] Drolet G.G., Huemmrich K.F., Hall F.G., Middleton E.M., Black T.A., Barr A.G., Margolis H.A. (2005). A MODIS-derived photochemical reflectance index to detect inter-annual variations in the photosynthetic light-use efficiency of a boreal deciduous forest. Remote Sens. Environ..

[85] Hilker T., Coops N.C., Nesic Z., Wulder M.A., Black A.T. (2007). Instrumentation and approach for unattended year round tower based measurements of spectral reflectance. Comput. Electron. Agric..

[86] Liu L., Zhang Y., Jiao Q., Peng D. (2013). Assessing photosynthetic light-use efficiency using a solar-induced chlorophyll fluorescence and photochemical reflectance index. Int. J. Remote Sens..

